# Enhanced oral delivery of insulin via the colon-targeted nanocomposite system of organoclay/glycol chitosan/Eudragit^®^S100

**DOI:** 10.1186/s12951-020-00662-x

**Published:** 2020-07-25

**Authors:** Sang Hoon Lee, Seung-Yun Back, Jae Geun Song, Hyo-Kyung Han

**Affiliations:** College of Pharmacy, Dongguk University-Seoul, Dongguk-ro-32, Ilsan-Donggu, Goyang, Korea

**Keywords:** Insulin, Oral delivery system, Colon targeting, Nano-carrier, Aminoclay

## Abstract

This study aimed to develop a ternary nanocomposite system of organoclay, glycol-chitosan, and Eudragit^®^S100 as an effective colon targeted drug delivery carrier to enhance the oral absorption of insulin. A nanocomplex of insulin and aminoclay was prepared via spontaneous co-assembly, which was then coated with glycol-chitosan and Eudragit S^®^100 (EGAC-Ins). The double coated nanocomplex, EGAC-Ins demonstrated a high entrapment efficiency of greater than 90% and a pH-dependent drug release. The conformational stability of insulin entrapped in EGAC-Ins was effectively maintained in the presence of proteolytic enzymes. When compared to a free insulin solution, EGAC-Ins enhanced drug permeability by approximately sevenfold in Caco-2 cells and enhanced colonic drug absorption in rats. Accordingly, oral EGAC-Ins significantly reduced blood glucose levels in diabetic rats while the hypoglycemic effect of an oral insulin solution was negligible. In conclusion, EGAC-Ins should be a promising colonic delivery system for improving the oral absorption of insulin.

## Introduction

Protein-based drugs cover diverse therapeutic indications and are beneficial in the treatment of many incurable diseases [1]. Particularly, the high target-selectivity of protein drugs can reduce undesirable side effects and toxicity [2]. Thus, a large amount of research and development has been devoted to biological drugs including proteins and peptides. In current medical practice, protein drugs are primarily administered via parenteral injection because of their low bioavailability [3]. Therefore, there is a high demand for the non-invasive formulations of protein drugs that can improve patient compliance.

Diabetes mellitus (DM) is a chronic metabolic disease that is present in a large portion of the patient population (i.e. more than 415 million adults), and it is predicted to reach 642 million patients by 2040 [4]. DM is classified into two main types: type 1 and type 2 [5]. Type 1 DM is caused by autoimmune reactions of beta cells in pancreatic islets [6]. Type 2 DM is caused by either malfunctioning or defective pancreatic beta cell that lead to reduced insulin signaling and secretion from the pancreas. Insulin is used to treat type 1 DM patients that exhibit insulin deficiency as well as type 2 patients at a later stage when adequate glycemic control is not achievable by diet, regular exercise, weight loss, or oral anti-diabetic agents [7, 8]. Since high blood glucose levels can damage tissues and cause severe complications including blindness, cardiovascular diseases, and non-traumatic amputation [4], effective insulin administration is important in the treatment of DM.

Insulin is primarily administered via subcutaneous (SC) injections [9]; however, injectable formulations can have drawbacks that include pain, immune reactions, infections, hypoglycemia, and the discomfort of long-term therapy [4, 9]. As a result, many researchers have tried to develop non-invasive insulin formulations via the alternative routes of administration such as oral, nasal, ocular, and transdermal. Oral administration is the most preferred route of administration because it is cost-effective, safe, and pain-free [4]. However, the oral delivery of insulin has many obstacles including physicochemical instability in the gastrointestinal tract, low intestinal permeability due to high molecular mass and hydrophilicity, and extensive enzymatic degradation that lead to extremely low oral bioavailability and ineffectiveness in controlling blood glucose level [10–12]. Therefore, many formulation approaches have attempted to improve the intestinal absorption as well as the physicochemical and enzymatic stability of orally administered insulin, which are exemplified by M-cell targeted- or colon-targeted delivery systems and cell-penetrating protein (CPP)-conjugated nanoparticles [13]. Particularly, colon-targeted protein drug delivery systems have been actively pursued because the colon has low proteolytic activity, has a neutral pH, is highly responsive to absorption enhancers, and allows for longer residence time [14, 15].

Aminoclay (3-aminopropyl functionalized magnesium phyllosilicate) is a nontoxic, silicate-based material that is positively charged in water [16, 17]. In addition, aminoclay has a high adsorption capacity [17, 18]. Glycol-chitosan is biocompatible and biodegradable, has low immunogenicity, and exhibits good aqueous solubility in all pH ranges [19–22]. Glycol-chitosan can retain its positive charge in the small intestine, which may enhance its retention in the GI tract via electrostatic interactions with negatively charged intestinal membranes [23, 24]. Since the solubility of Eudragit^®^ S100 is pH-dependent (i.e. it dissolves at pHs greater than 7.0) [9, 25, 26], an Eudragit^®^ S100 surface coating can prevent drug release in the stomach and upper intestine [27]. Therefore, the present study involved the construction of an insulin colonic delivery system comprised of an insulin and aminoclay nanocomplex coated with glycol-chitosan and Eudragit^®^ S100. The structural and in vitro characteristics of the developed nanoparticles were evaluated, and the in vivo effectiveness was assessed in diabetic rats.

## Results and discussion

### Structural characterization of nanoparticles

As summarized in Table 1, all nanoparticles had a narrow size distribution and a high entrapment efficiency greater than 90%. Insulin-aminoclay nanocomplex (AC-Ins), prepared via electrostatic interactions, was 192 ± 8.20 nm in size and had a zeta-potential of − 14.0 ± 4.4 mV. The sequential coating of AC-Ins with glycol-chitosan and Eudragit^®^ S100 increased the size of the nanoparticles and reversed their surface charge (particle size and zeta-potential were 319 ± 9.42 nm and 15.6 ± 2.31 mV for GAC-Ins and 412 ± 10.4 nm and − 20.6 ± 0.76 mV for EGAC-Ins).

**Table 1:** Characteristics of nanocomposite formulations (Mean ± SD, n = 3)

Formulation	Size (nm)	PDI	Zeta-potential (mV)	EE (%)
AC-Ins	192 ± 8.20	0.20 ± 0.01	− 14.0 ± 4.41	97.6 ± 1.20
GAC-Ins	319 ± 9.42	0.46 ± 0.02	15.6 ± 2.31	90.7 ± 3.14
EGAC-Ins	412 ± 10.4	0.32 ± 0.02	− 20.6 ± 0.76	90.3 ± 1.06

**Fig. 1:**
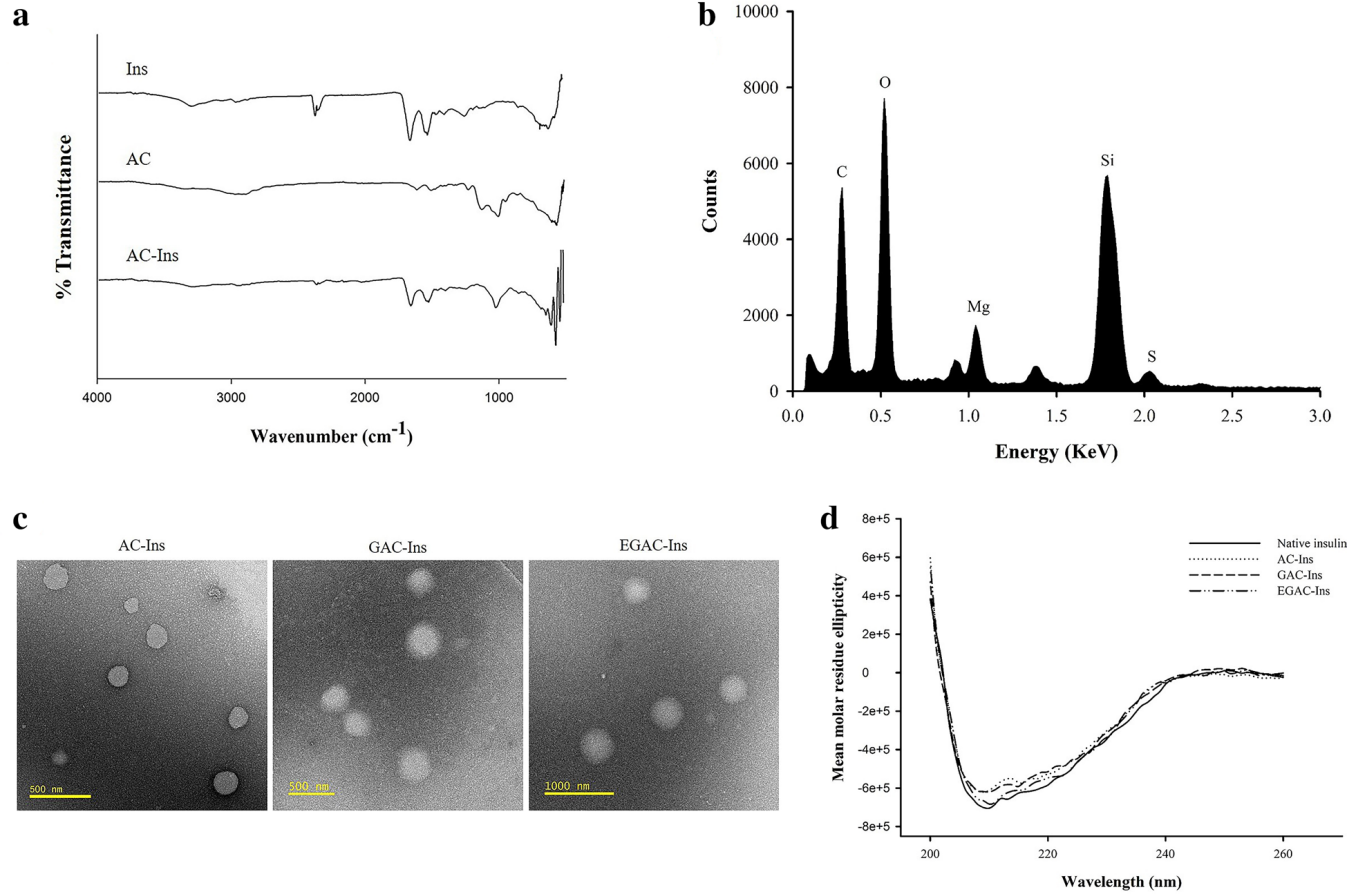
*In vitro* characterization of formulations. FT-IR spectra (**a**), EDX spectrum of EGAC-Ins (**b**), TEM images (**c**) (scale bar: 500 nm for AC-Ins and GAC-Ins, 1000 nm for EGAC-Ins), and CD spectra of insulin released from the nanoparticles (**d**)

The formation of AC-Ins was confirmed by FT-IR analysis. As shown in Fig. 1a, the FT-IR spectrum of aminoclay indicated the absorption bands of N–H (1608 cm^−1^, 1502 cm^−1^), Si–O-Si (995 cm^−1^), and Mg-O (550 cm^−1^) [16]. The FT-IR spectrum of insulin exhibited amide I peak at 1645 cm^−1^ and amide II peak at 1515 cm^−1^. The FT-IR spectrum of AC-Ins exhibited absorption bands from both insulin and aminoclay such as peaks at 1514 cm^−1^ and 1644 cm^−1^ from the amide bonds of insulin and at 1009 cm^−1^ and 550 cm^−1^ from aminoclay [28], suggesting the formation of an insulin and aminoclay complex. Furthermore, EDX analysis of EGAC-Ins indicated the elemental composition originating from aminoclay (Si, Mg) and insulin (S), confirming the integration of insulin and aminoclay into the nanoparticles (Fig. 1b). The morphology of the nanoparticles was also examined by TEM. As shown in Fig. 1c, the nanoparticles had spherical shapes and their size was comparable to the size determined by dynamic light scattering.

Since the structural stabilization of proteins is critical in the formulation development process of protein drugs [29], the conformational stability of insulin entrapped in the nanoparticles was evaluated by CD spectroscopy and compared with native insulin. As shown in Fig. 1d, the CD spectra of insulin released from the nanoparticles were similar to that of native insulin, indicating that the conformational structure of insulin was preserved in the nanoparticles.

### In vitro drug release studies

**Fig. 2:**
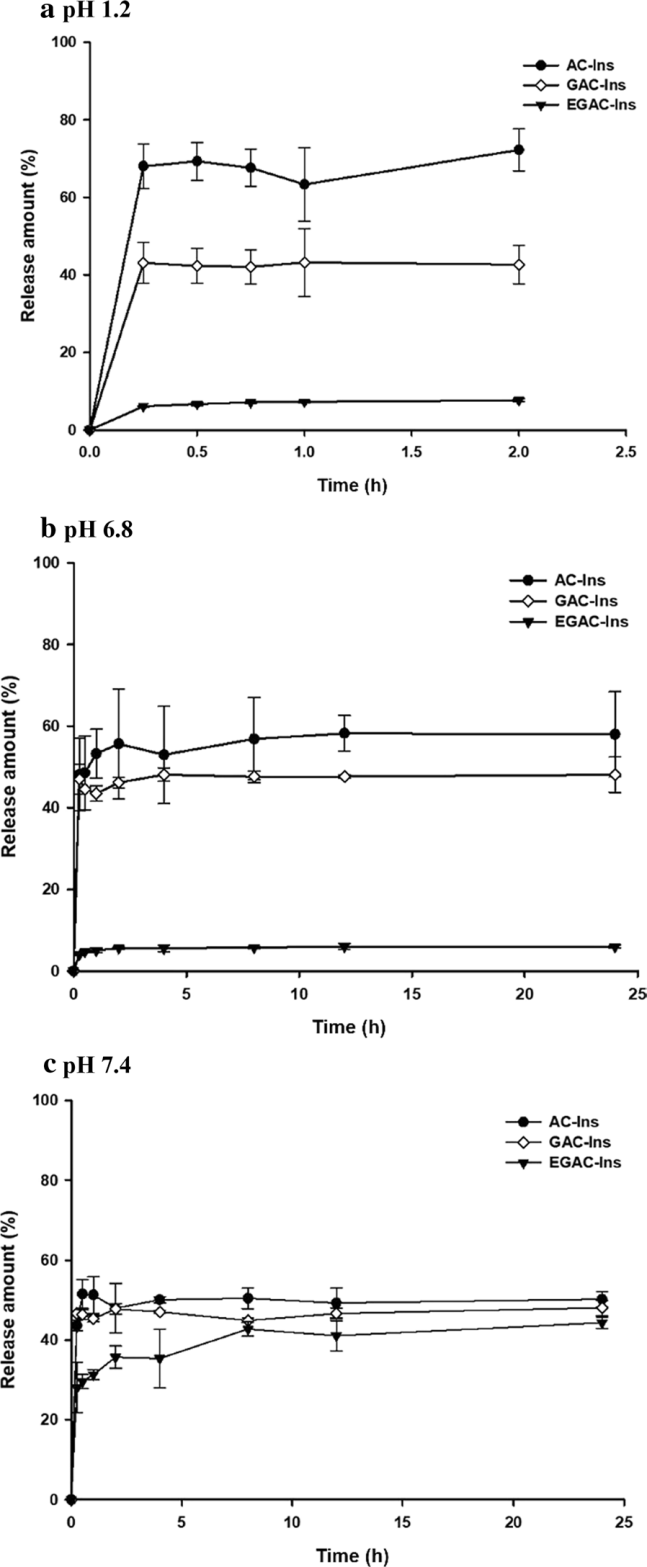
Drug release profiles of the nanoparticles at different pHs (mean ± SD, n = 3)

Drug release profiles of the nanoparticles were examined in a pH range of 1.2–7.4 to reflect intestinal pH conditions. As summarized in Fig. 2, AC-Ins exhibited rapid drug release between acidic to neutral pHs, and it released 60–70% of the drug within 15 min at pH 1.2. In contrast, EGAC-Ins showed minimal drug release (< 10%) at pH 1.2 and pH 6.8 because the outer coating layer of Eudragit^®^ S100 is insoluble at pH less than 7.0. Since Eudragit^®^ S100 has carboxyl groups that ionize when the pH changes from acidic to alkaline, the outer coat of Eudragit^®^ S100 dissolves at pH 7.4 [9, 25, 26, 30]. Accordingly, drug release from EGAC-Ins increased up to 40% at pH 7.4 and was similar to those from AC-Ins and GAC-Ins (Fig. 2). The pH-dependent drug release characteristics of EGAC-Ins could be beneficial because they can protect insulin from the acidic environment of the stomach and release it in the large intestine.

### Protection against enzymatic degradation

**Fig. 3:**
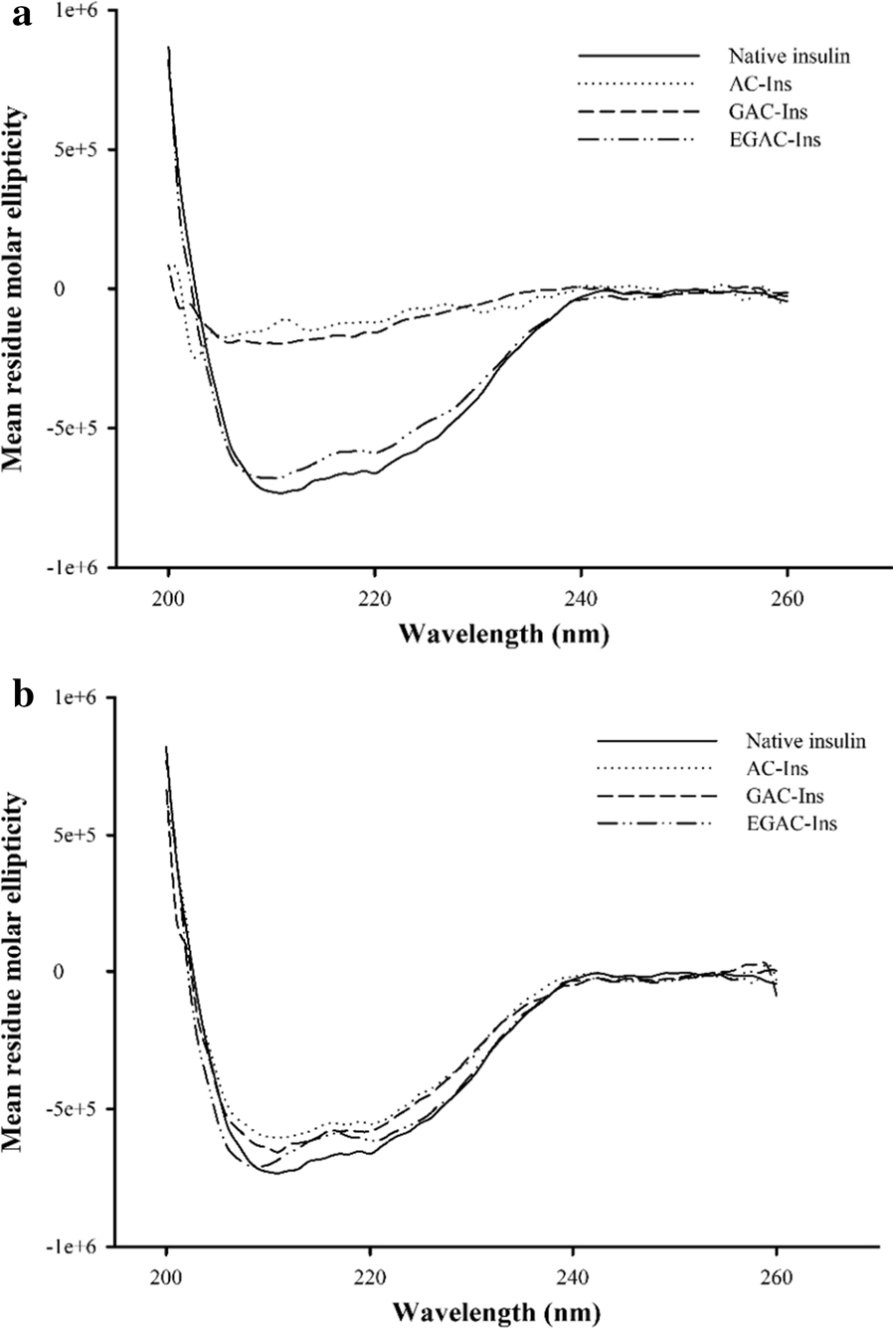
CD spectra of insulin released from different formulations after incubation in SGF for 1 h (**a**) and SIF for 2 h (**b**)

Protein drugs are susceptible to gastrointestinal (GI) destabilization by the acidic gastric fluids and proteolytic enzymes in the GI tract. This is a major barrier to oral protein delivery [31, 32]. Therefore, the protective effect of the nanoparticles against the gastrointestinal destabilization of insulin was examined by using simulated gastrointestinal fluids containing proteolytic enzymes. After incubating each formulation in SGF or SIF, the structural stability of insulin entrapped in the nanoparticles was evaluated by CD spectroscopy. As summarized in Fig. 3, insulin entrapped in AC-Ins and GAC-Ins was completely degraded in SGF. In contrast, insulin in EGAC-Ins retained its conformational stability in both SGF and SIF. These results suggest that EGAC-Ins are effective at protecting insulin against gastrointestinal destabilization.

### Cellular transport studies

Before the cellular uptake and transport studies, the cytotoxicity of the developed nanoparticles was evaluated via MTT studies. None of the nanoparticles were cytotoxic in Caco-2 cells up to a 100 µM drug concentration (data not shown).

**Fig. 4:**
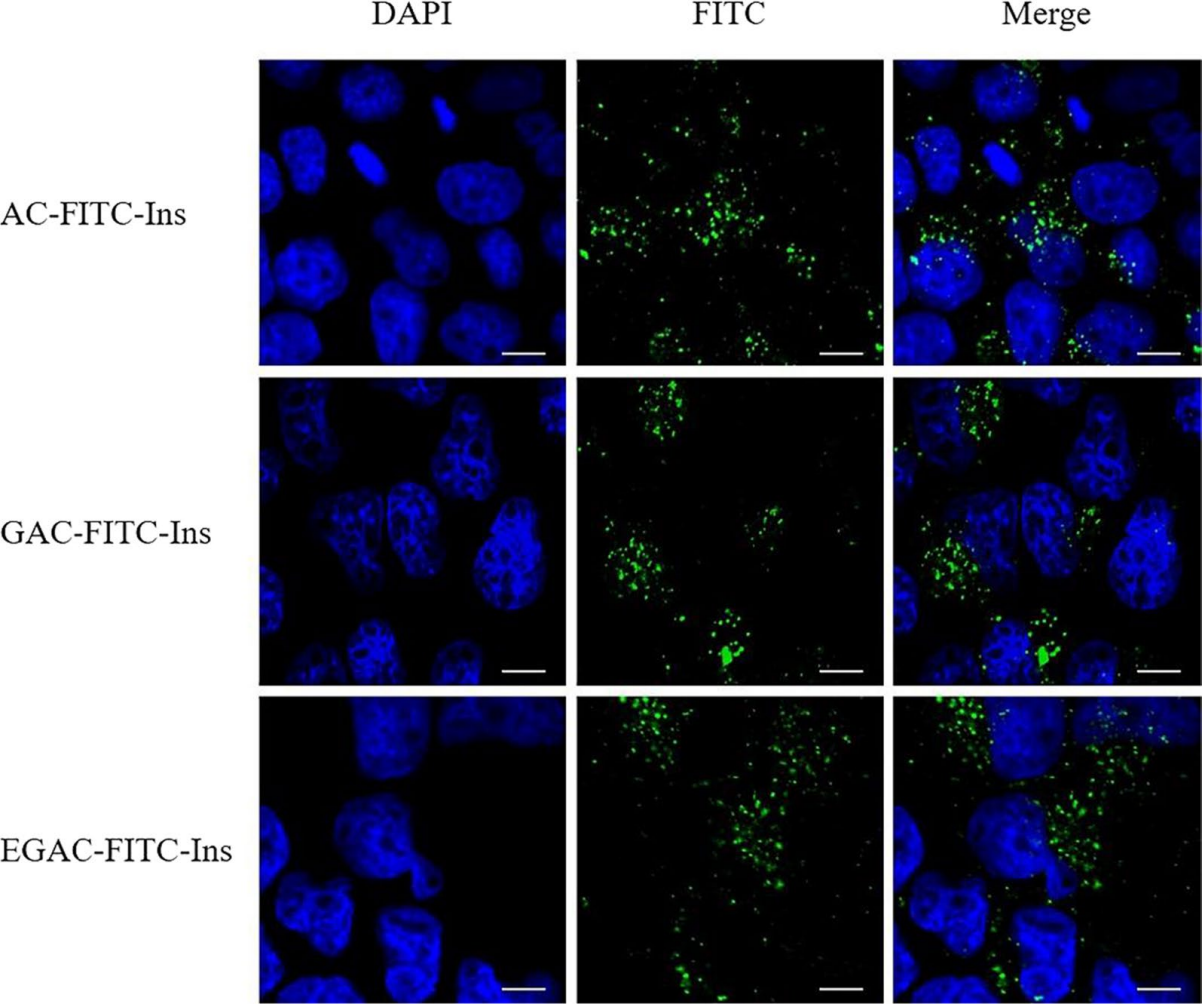
CLSM images showing the intracellular trafficking of FITC-Ins from different formulations in Caco-2 cells. The first panel shows DAPI-stained nuclei, and the second panel displays the green fluorescence produced by FITC. Scale bar represents 10 μm

**Fig. 5:**
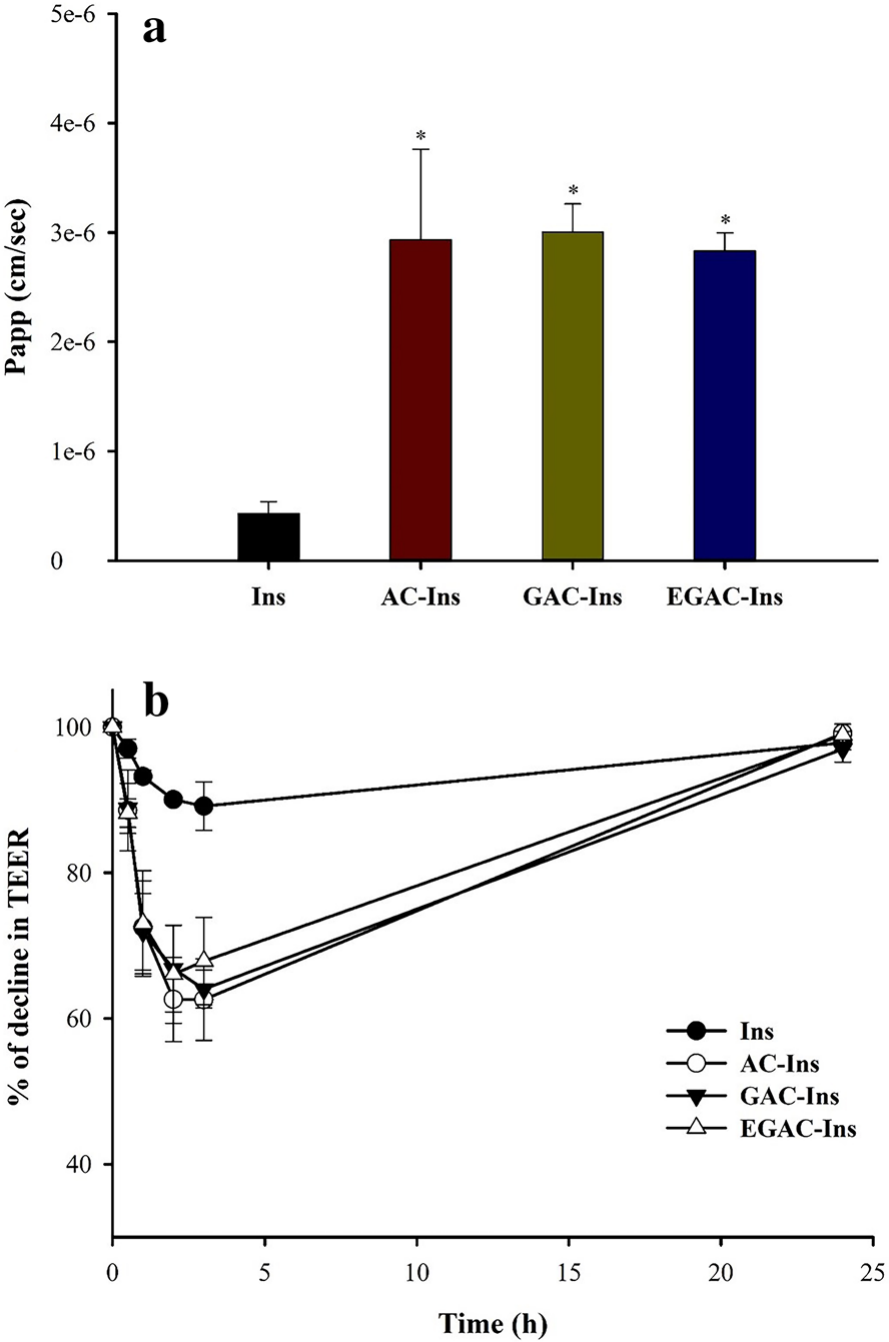
Cellular transport of insulin with different formulations (**a**), and the effect of nanoparticles on the trans-epithelial electrical resistance (TEER) (**b**) in Caco-2 cells (mean ± SD, n = 3). TEER values were measured during incubation in the presence and absence of nanoparticles. After 4 h of incubation, the drug solution was replaced by fresh culture medium, and TEER value recovery was monitored over time. *p <0.05, compared to free insulin solution

The cellular uptake of nanoparticles containing fluorescent labelled-insulin (FITC-Ins) was evaluated in Caco-2 cells. As illustrated in Fig. 4, a bio-imaging assay using CLSM showed the effective intracellular distribution of FITC-Ins by the nanocomposite formulations. After incubating cells with the nanoparticles, strong fluorescence intensity of FITC-Ins was observed in the cytoplasm around the DAPI-stained nuclei. This implies that the nanoparticles were effective at enhancing the intracellular uptake of insulin. In parallel, the apical to basolateral permeability (P_app_) of insulin using different formulations was also determined in Caco-2 cells. Compared to a free insulin solution, all of the nanoparticles increased drug permeability approximately sevenfold (Fig. 5a). This might have occurred, at least in part, because glycol-chitosan and aminoclay may facilitate the paracellular transport of nanoparticles via tight junction opening [24, 33]. Therefore, TEER values were measured during incubation in the presence and absence of the nanoparticles. As shown in Fig. 5b, TEER values significantly decreased in the presence of nanoparticles and fully recovered after the nanoparticles were removed. These results suggest that the nanoparticles likely have a reversible and transient effect on tight junction opening and enhance drug permeation.

Taken together, these results suggest that the nanoparticles might be effective at improving insulin transport across the epithelial membrane.

### Colonic absorption study in rats

**Fig. 6:**
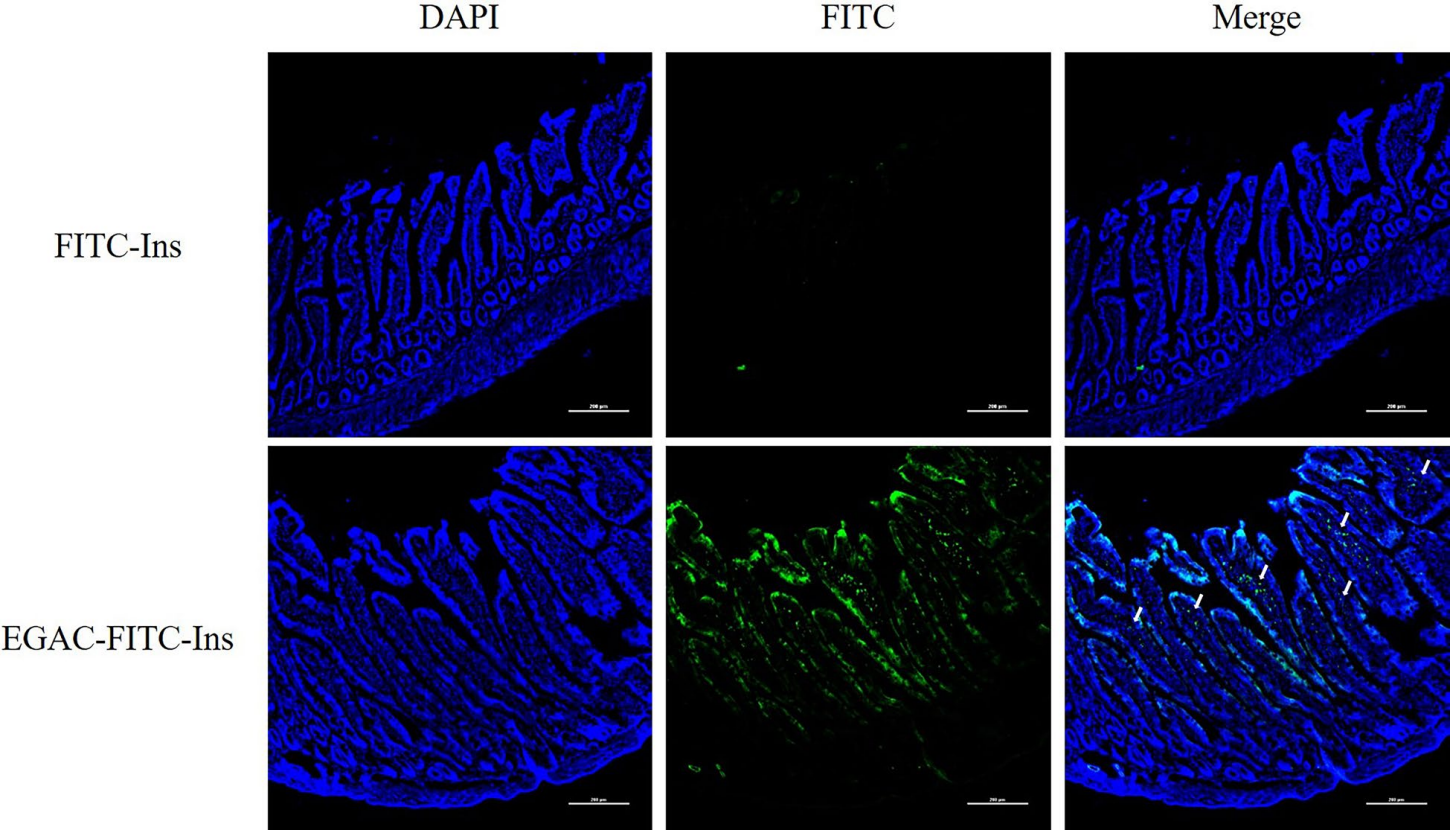
CLSM images of excised colon after oral administration of FITC-Ins or EGAC-FITC-Ins in rats. Dose was equivalent to 50 IU/kg FITC-Ins, and colonic sections were taken 6 h after dosage (scale bar represents 200 μm)

The effectiveness of EGAC-Ins as a colon-targeted drug delivery system was evaluated by monitoring colonic drug absorption in rats. After oral administration of EGAC-FITC-Ins or a free FITC-Ins solution, the extent of colonic drug permeation was examined with a bioimaging assay. As illustrated in Fig. 6, after oral administration of free FITC-Ins solution, fluorescence signals were negligible in the colonic tissues of rats. This is likely due to the physicochemical and enzymatic instability of insulin in the GI tract before reaching the colon as well as low drug permeability [38]. Alternatively, EGAC-FITC-Ins achieved significantly higher colonic distribution when compared to the free drug solution. Several factors may explain the enhanced colonic drug distribution via EGAC-FITC-Ins. First, the pH-dependent polymer coating may minimize premature drug release in the upper GI tract and protect the entrapped insulin from acidic and proteolytic degradation. Second, after dissolution of the Eudragit S100 coating layer in the colon, the released cationic nanoparticles might interact with negatively charged colonic mucosa and increase drug residence time in the colon. Third, based on in vitro studies in Caco-2 cells, the nanoparticles may enhance the drug permeation across the epithelial membrane.

Collectively, EGAC-FITC-Ins was more effective at facilitating colonic insulin absorption in rats, when compared to free drug solution.

### Hypoglycemic effect in diabetic rats

**Fig. 7:**
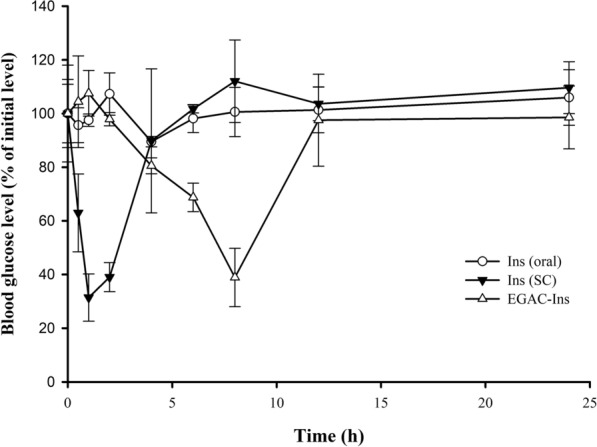
Hypoglycemic effect after oral or subcutaneous injection of each formulation in diabetic rats (mean ± SD, n = 3)

The hypoglycemic effect of orally-administered nanoparticles and conventional SC injection was compared in diabetic rats. As summarized in Fig. 7, SC injection of insulin solution rapidly decreased blood glucose concentrations by up to 31.4% of initial blood glucose levels within 1 h, which was maintained for 1 h and then gradually recovered to initial blood glucose levels within 6 h after dosage. In contrast, orally administered insulin solution did not exhibit any hypoglycemic effect, likely due to instability in the GI tract and low membrane permeability. After oral administration of EGAC-Ins, blood glucose levels significantly decreased by up to 38.9% of initial blood glucose concentrations within 8 h and then gradually recovered to initial blood glucose levels within 12 h after dosage. Consistent with the in vitro studies, the improved stability and permeability of insulin delivered via EGAC-Ins nanoparticles significantly enhanced the hypoglycemic effect of orally administered insulin. When compared to SC injection, orally administered EGAC-Ins exhibited a slower and longer duration of action. EGAC-Ins minimized burst drug release in the stomach and upper intestine and slowly released insulin in the lower intestine and colon. Therefore, the onset time of orally administered EGAC-Ins was longer than that from SC injection of insulin solution [39]. However, it lowered blood glucose levels for a longer time period when compared to SC injection.

Collectively, these results suggest that EGAC-Ins is an effective oral delivery system of insulin.

## Conclusion

In this study, a colon-targeted ternary nanocomposite system composed of organoclay/glycol-chitosan/Eudragit^®^ S100 was developed to enhance the oral delivery of insulin. EGAC-Ins, an insulin-loaded ternary nanocomposite formulation, was obtained as nano-sized particles with high entrapment efficiency (> 90%). EGAC-Ins displayed pH-dependent drug release characteristics that minimized premature drug release in the upper GI tract. The conformational stability of insulin entrapped in EGAC-Ins was preserved in simulated gastrointestinal fluids. In addition to the enhanced cell permeability and colonic absorption of insulin in rats via EGAC-Ins, orally-administered EGAC-Ins significantly improved the hypoglycemic effect in diabetic rats while the efficacy of oral insulin solution was negligible. Taken together, the present study suggests that EGAC-Ins might be useful to enhance the bioavailability and efficacy of oral insulin.

## Methods

### Materials

Insulin was purchased from FUJIFILM Wako Pure Chemical Co. (Osaka, Japan). Eudragit^®^S100 was obtained from Evonik Korea Ltd. (Seoul, Korea). Glycol-chitosan, fluorescein isothiocyanate (FITC)-labeled insulin, formic acid, pepsin, trypsin, and 3-aminopropyltriethoxysilane (APTES, 99%) were purchased from Sigma-Aldrich Co. (St Louis, MO, USA). Dulbecco’s Modified Eagle’s medium (DMEM), Hank’s balanced salt solution (HBSS), non-essential amino acids, fetal bovine serum (FBS), penicillin–streptomycin, and all other reagents used in cell culture studies were obtained from GE Healthcare Life Sciences (South Logan, UT, USA). 4′,6-diamidino-2-phenylindole (DAPI) was purchased from Invitrogen Molecular Probes (Karlsruhe, Germany). Magnesium chloride hexahydrate (98%) and other inorganic salts were purchased from Junsei Chemical Co., Ltd (Tokyo, Japan). All other chemicals and reagents were HPLC-grade.

Caco-2 cells (human epithelial colorectal adenocarcinoma cells) were purchased from the Korean Cell Line Bank (Seoul, Korea). Cells were grown in DMEM containing 10% FBS, 1% nonessential amino acid, and 1% antibiotics. Cells were incubated at 37 °C in an atmosphere of 5% CO_2_ and 90% relative humidity.

### Preparation of nanoparticles

First, 3-aminopropyl-functionalized magnesium phyllosilicate (aminoclay) was synthesized by a previously described method [16]. Briefly, magnesium chloride (8.4 g) was dissolved in ethanol (200 mL). 3-Aminopropyltriethoxysilane (13 mL) was then added dropwise under vigorous stirring at 200 rpm, which rapidly formed white precipitate that was stirred overnight. The resulting product was separated by centrifugation, washed three times in ethanol, and dried in an oven at 50 °C. Aminoclay was exfoliated by dispersing the bulk powder in water followed by ultra-sonication for 10 min.

An insulin-aminoclay nanocomplex (AC-Ins) was prepared by electrostatic interactions between aminoclay and insulin. Briefly, insulin (10 mg) was dissolved in 10 mM HCl (1 mL), and 100 mM Tris–HCl was added at a ratio of 1:1 (v/v). This insulin solution (5 mg/mL) was added dropwise into an aqueous suspension of aminoclay (10 mg/mL) at a drug/clay ratio of 1:3 at room temperature. After stirring for 4 h, the generated AC-Ins was separated by centrifugation (22,250*g*) at 4 °C for 10 min and air-dried at room temperature. The aqueous suspension of AC-Ins (3 mg/mL) was added dropwise into an equal volume of a 0.2% glycol-chitosan solution at pH 5.5 while stirring at 300 rpm for 30 min. The resultant nanoparticles (GAC-Ins) were collected by ultra-centrifugation (77,100*g*) at 4 °C for 15 min. After re-dispersion of GAC-Ins in distilled water, the GAC-Ins aqueous suspension (3 mg/mL) was added into an equal volume of 0.2% Eudragit^®^ S100 dissolved in ethanol while stirring at 300 rpm for 30 min. After ultra-centrifugation (77,100*g*) at 4 °C for 15 min, the collected nanoparticles (EGAC-Ins) were air-dried at room temperature.

For the bioimaging study, FITC-insulin-loaded nanoparticles were also prepared using the same procedure described above; however, insulin was replaced by fluorescent FITC-insulin.

### Structural characterization of the nanoparticles

The particle size and zeta potential of all insulin-loaded nanoparticles were measured by dynamic light scattering (DLS) using a Zetasizer Nano-ZS90 (Malvern Instruments, Malvern, UK). The polydispersity index (PDI) was also measured as a dimensionless number indicating the size distribution. The entrapment efficiency (EE) of each nanoparticle was calculated by the following equation:

$${\text{EE }}\left( {\text{\%}} \right) = \frac{{{\text{Amount of drug initially added}} - {\text{Amount of drug in supernatant}}}}{\text{Amount of drug initially added}} \times 100$$

The structural characterization of all nanoparticles was also performed using Fourier transform infrared spectroscopy (FT-IR) (Nicolet™ iS™ 5; Thermo Fisher Scientific, Waltham, MA, USA) with a ZnSe crystal accessory. The FT-IR spectrum of each sample was measured over a wavenumber range of 4000–500 cm^−1^ with 64 scans at resolution of 4 cm^−1^.

Circular dichroism (CD) analysis was used to examine the structural stability of insulin released from the nanoparticles. Far UV CD spectra were collected using the Chirascan™-Plus Spectrometer (Applied Photophysics, Surrey, UK). Wavelength spectra were collected from 200 nm to 260 nm at 25 °C with a bandwidth of 1 nm and a light path length of 0.5 mm.

The morphological characteristics of the nanoparticles were monitored by transmission electron microscopy (TEM) (JEM-F200; JEOL Ltd., Tokyo, Japan). The compositional elements of the developed formulations were analyzed by energy-dispersive X-ray spectroscopy (EDX). TEM and EDX analyses were performed at the National Center for Inter-University Research Facilities (NCIRF) at Seoul National University (Seoul, Korea).

### In vitro drug release studies

The drug release profiles of the nanoparticles were evaluated at pHs 1.2, 6.8, and 7.4. Nanoparticles (equivalent to 0.2 mg/mL insulin) were dispersed in release medium, while shaking at 100 rpm at 37 °C. At predetermined time points, samples were collected and centrifuged at 22,250*g* for 10 min. The supernatants were analyzed by HPLC to determine the amount of drug released. CD analysis was also used to detect the molecular conformation of insulin released from the nanoparticles.

### Protection against enzymatic degradation

To examine the protective effect of the nanoparticles against proteolytic degradation, the structural stability of insulin entrapped in the nanoparticles was evaluated in simulated gastric juice (SGF, pH 1.2 with 5 µg/mL pepsin) and intestinal fluid (SIF, pH 7.4 with 50 µg/mL trypsin) as reported by Rekha and Sharma [40] with slight modifications. An aliquot of nanoparticles was incubated in SGF (1 mL) or SIF (1 mL) at 37 °C. At designated time points, the enzymatic reaction was terminated by the addition of either 0.2 mL of 0.2 M NaOH into SGF or 0.1 M HCl into SIF. The nanoparticles were collected by centrifugation at 22,250*g* for 10 min, and insulin in the nanoparticles was extracted in phosphate buffer (pH 7.4) for 2 h. The conformational stability of insulin extracted from the nanoparticles was examined by CD analysis.

### Confocal laser scanning microscopy (CLSM)

The intracellular localization of the nanoparticles was examined by confocal laser scanning microscopy (CLSM). Briefly, cells were seeded on slides in 24-well plates at a density of 5×10^4^ cells per well and incubated for 24 h. After removing the medium, the cells were washed three times with PBS. Each formulation (FITC-Ins, AC-FITC-Ins, GAC-FITC-Ins, or EGAC-FITC-Ins) in HBSS was added to the cells at a concentration equivalent to 25 µg/mL FITC-Ins and incubated at 37 °C. After incubating for 3 h, the cells were washed three times with PBS and fixed in 4% paraformaldehyde for 15 min. The nuclei were stained with DAPI for 3 min at room temperature. The intracellular distribution of nanoparticles was visualized using a Nikon C1 CLSM with EZ-C1 software (Nikon, Tokyo, Japan).

### Trans-epithelial transport studies

Caco-2 cells were seeded at a density of 2.0 × 10^5^ cells/well on trans-well plates with a surface area of 1.12 cm^2^. The cells were incubated at 37 °C for 21 days, and the medium was refreshed regularly. The integrity of cell monolayers was monitored by measuring trans-epithelial electrical resistance (TEER) using an epithelial tissue voltohmmeter (Millicell ERS-2, Merck KGaA, Darmstadt, Germany).

Prior to the transport studies, the medium was replaced with HBSS and incubated at 37 °C for 30 min. Then, HBSS on the apical side was replaced by a drug solution containing each formulation equivalent to 10 µg/mL insulin. At predetermined time points, samples were collected from the basolateral compartment and replenished with an equal volume of fresh HBSS to maintain a constant volume. Drug concentrations were determined using an LC–MS/MS. The apparent permeability coefficient (P_app_) was calculated using the following equation: P_app_ = dQ/dt × 1/AC_0_, where dQ/dt is the cumulative drug amount in the basolateral compartment as a function of time, A is the surface area of membrane filter, and C_0_ is the initial drug concentration in the apical compartment.

### Ex vivo permeation studies in rats

Animal studies were conducted in accordance with the “Guiding Principles in the Use of Animals in Toxicology” adopted by the Society of Toxicology (USA), and the study protocol was approved by the review committee of Dongguk University (IACUC-2017-016-4). Male Sprague–Dawley rats (150–170 g) were obtained from Orient Bio Inc. (Seongnam, Korea). Rats were fasted for 12 h before the experiment but were given free access to tap water. An aqueous suspension of EGAC-FITC-Ins or FITC-Ins was administered to the rats by oral gavage at a dose equivalent of 50 IU/kg FITC-Ins. Rats were sacrificed 6 h after dosage, and colons were excised immediately after laparotomy. The colons were washed with PBS to remove the luminal content. The colon tissues were fixed with 4% paraformaldehyde for 1 day and placed in 30% sucrose solution until the tissues sank. Next, the colon tissues were cryo-sectioned and examined by confocal microscopy (Nikon C1, Nikon, Tokyo, Japan) to visualize the localization of FITC-Ins.

### Hypoglycemic effect in diabetic rats

Male Wistar rats (150 ± 20 g) were supplied by Orient Bio Inc. (Seongnam, Korea). The study protocol was approved by the review committee of Dongguk University (IACUC-2017-016-4). Rats were fasted for 12 h before the experiment but were allowed water ad libitum. Diabetes was induced by an intraperitoneal injection of streptozotocin (STZ in 0.05 mol/L citrate buffer solution, pH 4.5) at a dose of 65 mg/kg. After 4 days, blood was collected from tail veins, and blood glucose levels were measured with a Roche glucose meter (ACCU-CHEK^®^ guide). Rats with a fasting blood glucose over 250 mg/dL were used as diabetic rat models [41].

Twelve STZ-induced diabetic rats were randomly divided into 3 groups. All rats were fasted for 6 h before the experiment but were allowed water ad libitum. Group 1 was administered an insulin solution (5 IU/kg) via SC injection. Groups 2 and 3 were administered an insulin solution or EGAC-Ins, respectively, at the dose equivalent of 100 IU/kg of insulin via oral gavage. At predetermined time points, blood samples were collected from tail veins to measure blood glucose levels. Blood glucose levels were measured using a Roche glucose meter (ACCU-CHEK^®^ guide) and were expressed as a percentage of the initial glucose levels [9].

### Analytical assays

Drug concentrations were determined by HPLC and LC–MS/MS. The HPLC system (Flexar; Perkin Elmer, MA, USA) consisted of an automatic injector, a UV detector and two solvent delivery pumps. Samples were injected into the HPLC system connected to a column (Gemini C18, 4.6 × 150 mm, 5 μm; Phenomenex, Torrance, CA, USA). Chromatographic separation was achieved by eluting the mobile phase (acetonitrile: 0.1% trifluoroacetic acid = 30:70, v/v) at a flow rate of 1 mL/min with a column temperature of 30 °C. Prednisone was used as an internal standard. The detection wavelength was set to 215 nm. The calibration curve was obtained in the range of 10–500 µg/mL with good linearity (R^2^ > 0.9996).

For LC–MS/MS analysis, the chromatographic separation of analytes was performed with a C18 column (4.6 × 100 mm, 2.6 μm; Phenomenex, Torrance, CA, USA) at 30 °C. The mobile phase consisted of acetonitrile with 0.1% formic acid and water with 0.1% formic acid (60:40, v/v), and the flow rate was 1 mL/min. Mass spectrometric detection was achieved using positive ion electrospray ionization in multiple-reaction monitoring (MRM) mode by an ABSciex API 4000 triple quadrupole mass spectrometer (ABSciex, Framingham, MA, USA). The precursor/product ion pair (m/z) was 1162.6/143.2 for the drug and 271.2/155.1 for the internal standard (tolbutamide). The calibration curve was obtained in the range of 20–1000 ng/mL with good linearity (R^2^ > 9996).

### Statistical analyses

All data are expressed as the mean ± standard deviation (SD). Statistical analyses were performed using one-way ANOVA followed by Dunnett’s test. Values of *p *< 0.05 were considered significant.
